# Gastrointestinal microbial populations can distinguish pediatric and adolescent Acute Lymphoblastic Leukemia (ALL) at the time of disease diagnosis

**DOI:** 10.1186/s12864-016-2965-y

**Published:** 2016-08-15

**Authors:** Seesandra V. Rajagopala, Shibu Yooseph, Derek M. Harkins, Kelvin J. Moncera, Keri B. Zabokrtsky, Manolito G. Torralba, Andrey Tovchigrechko, Sarah K. Highlander, Rembert Pieper, Leonard Sender, Karen E. Nelson

**Affiliations:** 1 J. Craig Venter Institute (JCVI), 9714 Medical Center Drive, Rockville, MD 20850 USA; 2 JCVI, La Jolla, 92037 CA USA; 3 Hyundai Cancer Genomics Center, Children’s Hospital Orange County (CHOC Children’s), Orange, CA USA; 4 Division of Hematology-Oncology, Department of Medicine, School of Medicine, University of California-Irvine, Orange, CA USA; 5 Division of Oncology, Hyundai Cancer Institute, CHOC Children’s, Orange, CA USA; 6 Department of Pediatrics, School of Medicine, University of California-Irvine, Orange, CA USA

**Keywords:** Pediatric leukemia, Gastrointestinal microbiota, 16S rRNA gene sequencing, rRNA, Ribosomal RNA

## Abstract

**Background:**

An estimated 15,000 children and adolescents under the age of 19 years are diagnosed with leukemia, lymphoma and other tumors in the USA every year. All children and adolescent acute leukemia patients will undergo chemotherapy as part of their treatment regimen. Fortunately, survival rates for most pediatric cancers have improved at a remarkable pace over the past three decades, and the overall survival rate is greater than 90 % today. However, significant differences in survival rate have been found in different age groups (94 % in 1–9.99 years, 82 % in ≥10 years and 76 % in ≥15 years). ALL accounts for about three out of four cases of childhood leukemia. Intensive chemotherapy treatment coupled with prophylactic or therapeutic antibiotic use could potentially have a long-term effect on the resident gastrointestinal (GI) microbiome. The composition of GI microbiome and its changes upon chemotherapy in pediatric and adolescent leukemia patients is poorly understood. In this study, using 16S rRNA marker gene sequences we profile the GI microbial communities of pediatric and adolescent acute leukemia patients before and after chemotherapy treatment and compare with the microbiota of their healthy siblings.

**Results:**

Our study cohort consisted of 51 participants, made up of matched pediatric and adolescent patients with ALL and a healthy sibling. We elucidated and compared the GI microbiota profiles of patients and their healthy sibling controls via analysis of 16S rRNA gene sequencing data. We assessed the GI microbiota composition in pediatric and adolescent patients with ALL during the course of chemotherapy by comparing stool samples taken before chemotherapy with stool samples collected at varying time points during the chemotherapeutic treatment. The microbiota profiles of both patients and control sibling groups are dominated by members of *Bacteroides*, *Prevotella*, and *Faecalibacterium*. At the genus level, both groups share many taxa in common, but the microbiota diversity of the patient group is significantly lower than that of the control group. It was possible to distinguish between the patient and control groups based on their microbiota profiles. The top taxa include *Anaerostipes*, *Coprococcus*, *Roseburia*, and *Ruminococcus2* with relatively higher abundance in the control group. The observed microbiota changes are likely the result of several factors including a direct influence of therapeutic compounds on the gut flora and an indirect effect of chemotherapy on the immune system, which, in turn, affects the microbiota.

**Conclusions:**

This study provides significant information on GI microbiota populations in immunocompromised children and opens up the potential for developing novel diagnostics based on stool tests and therapies to improve the dysbiotic condition of the microbiota at the time of diagnosis and in the earliest stages of chemotherapy.

**Electronic supplementary material:**

The online version of this article (doi:10.1186/s12864-016-2965-y) contains supplementary material, which is available to authorized users.

## Background

Acute lymphoblastic leukemia (ALL) is a malignant disease of the bone marrow in which early lymphoid precursors proliferate and replace the normal hematopoietic cells of the marrow [1]. The lymphoid progenitor cells in patients with ALL are affected by the disease, leading to an impaired immune system typically observed at the time of diagnosis. ALL is the most common type of leukemia in children in the United States accounting for 26 % of all cancers in children up to 14 years of age and for 75 % of all pediatric leukemia cases [2]. All children and adolescents diagnosed with ALL will undergo chemotherapy as part of their treatment plan, and their health can be severely compromised due to the treatment with chemotherapeutic drugs. Chemotherapy can damage healthy cells in the lining of the digestive system and gastrointestinal (GI) disturbances are often induced in response to chemotherapy. Chemotherapeutic and antibiotic treatment has a detrimental impact on the host microbial ecosystem, which is essential for host mucosal protection [3]. The cytotoxic effects of these treatments lead to additional immunosuppression, which entails episodes of febrile neutropenia and potentially life-threatening bloodstream infections. Further, use of prophylactic and therapeutic antibiotics disrupts the GI microbiome’s ecological balance [4]. Medium-term gastrointestinal health outcomes of chemotherapy and disruptions of the intestinal microbial ecology include vomiting, diarrhea, constipation and *Clostridium difficile* infection-associated diarrhea (CDAD). To varying degrees, survivors of children and adolescents treated for cancer experience a range of long-term growth and developmental, organ function, fertility and reproduction and psychological adverse outcomes. The growing population of survivors of pediatric cancer reflects a highly vulnerable group of individuals who will probably experience adverse health-related and quality-of-life outcomes during their subsequent lifetimes, as a result of their curative cancer treatment [5–8]. There is increasing evidence that the composition of the GI microbiome may affect, and is modulated by, the human immune system [9]. Perturbed GI microbiomes have been associated with decreased immune competence, detrimental metabolic changes (e.g. obesity and malnutrition), susceptibility to GI infections and inflammatory syndromes [10, 11]. Likewise, several microbiome studies have examined the impact of antibiotics on the microbiota of individuals using 16S rRNA gene sequencing alone [12–16] or combined with metatranscriptomics [17]. These studies have demonstrated that administration of antibiotics can perturb this microflora temporarily and in certain cases permanently. Antibiotic-mediated alteration of the gut microbiome can converts the global metabolic profile to one that favors *C. difficile* germination and growth [18]. Furthermore, chemotherapeutics have a detrimental effect on the intestinal microbial composition, coinciding in time with the development of chemotherapy-induced mucositis [19]. There is, however, a limited understanding of the GI microbiome composition of pediatric and adolescent leukemia patients and the impact, if any, of contemporary chemotherapeutic treatments. The rationale for this study is to define and compare the GI microbiota composition of pediatric and adolescent leukemia patients with their healthy sibling controls via analysis of high-throughput sequencing data. And also, to assess the changes in microbiota structure of pediatric and adolescent leukemia patients during chemotherapy by comparing the samples taken before and after chemotherapy at varying time points during the chemotherapeutic treatment. Our results provide significant information on GI microbiota composition in immunocompromised children and indicate that the baseline microbiota of immunocompromised children was substantially different from their healthy siblings. It creates the potential for the better management of GI and systemic complications associated with immunodeficiency and other disease conditions of this type. Furthermore, characterizing the GI microbiota dynamics following chemotherapy treatment will address what alterations happen to the GI microbiota during and following chemotherapy regimens and can correlate with response to treatment.

## Methods

### Study population

A cohort study was designed to assess the impact of chemotherapy on the GI microbiota of pediatric and adolescent patients diagnosed with acute B-cell leukemia. The study cohort consisted of 51 participants, made up of 23 matched patients and a healthy sibling and five unmatched patients. Five patients who did not have enrolled healthy siblings were also included in the cohort. Three subjects not complete the study: two withdrew and one subject was deceased. Subject demographics by age and gender are shown in Table 1. All study participants were enrolled in the Hyundai Cancer Institute, Children’s Hospital Orange County (CHOC Children’s), California, USA. Human subject protocol and consent forms were established, and approved by the Institutional Review Boards at CHOC Children’s and the J. Craig Venter Institute (JCVI). Stool samples were collected at the completion of each treatment stage during the patient's stay at the hospital, referred to as “sampling visits”. Samples marked “visit 1” were collected at the time of diagnosis before any chemotherapy was administered, and thus provided baseline microbiota for each patient. As such, patient’s samples were collected before chemotherapy, during induction chemotherapy (chemotherapy given to induce a remission), consolidation chemotherapy (chemotherapy given once a remission is achieved) and during maintenance therapy (chemotherapy given in lower doses to assist in prolonging a remission). All healthy sibling controls were sampled once, aligning with the time period before chemotherapy began on the patient (Additional file 1: Table S1), however, four siblings samples were collected at two time points, which were excluded from the analysis. All patients with ALL enrolled in the study received antibiotic prophylaxis with sulfamethoxazole and trimethoprim during treatment and steroid prophylaxis at the induction stage. Incidental use of antibiotics and occurrence of infections in the month before each visit were recorded. Additional file 1: Table S1 provides details of the sampling visits for each patient over the period of enrollment.

**Table 1:** Characteristics of study subjects

Diagnosis	Acute lymphocytic leukemia (total *n* = 28)		
Age group	Ages 0–14 (*n* = 21)	Ages 15–19 (*n* = 4)	Ages 20–23 (*n* = 3)
Gender	Male: 13 (62 %)	Male: 4 (100 %)	Male: 2 (67 %)
	Female: 8 (38 %)	Female: 0 (0 %)	Female: 1 (33 %)
Sibling	Healthy control (total *n* = 23)	Ages 2–25 years (median age 9.3 years)	
Gender	Male: 7 (30 %), Female: 16 (70 %)		

Three participants in the 20–23 years age group did not complete the study. Two withdrew, and one was deceased

### Sample collection

Samples were collected using the Human Microbiome Project (HMP) collection protocol section 7.3.3. with no modifications. Stool specimens were collected and transported to CHOC Children’s for deoxyribonucleic acid (DNA) extraction [20].

### DNA extraction

Bacterial DNA was extracted from the stool samples using the PowerSoil® DNA Isolation Kit from MO BIO Laboratories, Inc. (catalog no: 12888) and by using the protocol as described in Yooseph et al., [21].

### Library construction and sequencing

DNA was amplified using primers that targeted the V1-V3 regions of the 16S rRNA gene [22]. These primers included the i5 and i7 adaptor sequences for Illumina MiSeq sequencing as well as unique 8 bp indices incorporated onto both primers such that each sample received its own unique barcode pair. This method of incorporating the adaptors and index sequences onto the primers at the polymerase chain reaction (PCR) stage provided minimal loss of sequence data when compared to previous library construction methods that would ligate the adaptors to every amplicon after amplification. This method also allows generating sequence reads which were all in the same 5′-3′ orientation. Using approximately 100 ng of extracted DNA, the amplicons were generated with Platinum Taq polymerase (ThermoFisher, catalog no: 11304-011) and by using the following cycling conditions: 95 °C for 5 min for an initial denaturing step followed by 95 °C for 30 s, 55 °C for 30 s, 72 °C for 30 s for a total of 35 cycles followed by a final extension step of 72 °C for 7 min then stored at 4 °C. Once the PCR for each sample was completed, the amplicons were purified using the QIAquick PCR purification kit (QIAGEN, catalog no: 28104), quantified using Tecan fluorometric methods (Tecan Group, Männedorf, Switzerland), normalized, and then pooled in preparation for cluster generation followed by Illumina MiSeq sequencing using the dual index 2x300 bp format (Roche, Branford, CT) following the manufacturer’s protocol.

### 16S rRNA sequence data processing

After primer trimming, the paired-end reads were quality trimmed using the DynamicTrim program (available in the SolexaQA suite [23]). Subsequently, mothur (v.1.34.4) [24] was used to merge overlapping forward and reverse reads to generate contig sequences from the paired-end reads. Chimeric sequences were identified using the UCHIME [25] implementation in mothur; these sequences were removed from the downstream analysis. The resulting sequence set was clustered at different sequence identity thresholds (90, 95 and 97 %) using CD-HIT [26] to generate Operational Taxonomic Units (OTUs); OTU representatives were assigned taxonomy using the mothur implementation of the Ribosomal Database Project (RDP) Classifier [27]. While the RDP classifier has a goal of generating genus-level taxonomic assignments, not all sequences could, however, be confidently assigned taxonomy to the genus level (using the bootstrap confidence threshold of 80 %); we denote these sequences by appending the tag *unclassified* to the end of their taxonomic assignment (one of phylum, class, order, or family levels).

### Electronic medical records

Study-specific participant data was collected at each specimen collection time point. Also, multiple time-stamped data points on each participant starting from the initial visit were gathered in the electronic medical record (EMR) at CHOC Children’s. Events recorded in the EMR were obtained for each patient identified by anonymized participant identification numbers.

### 16S rRNA data analysis

The Shannon diversity index was calculated from the OTUs in the samples to assess the alpha diversity of the microbial communities they represent. This was done using mothur [24]. Calculations were performed on sub-sampled sequences to account for differences in sequencing depth across the samples. To test whether the microbiota diversity difference between the patients and controls was statistically significant, we applied the Wilcoxon Rank Sum test in R, the statistical programming language, [28] to calculate the *p*-value for the comparison. The mean age difference between patient and control groups was evaluated using the *t*-test. For the average microbiota diversity between different time points, we used a paired (one sample) *t*-test.

The signature associated with the patient and control groups were identified using Random Forests (RF) [29], as implemented in the RF package (version 4.6–10) that is available in the R programming language and environment for statistical computing (version 3.2.2) [28]. For this analysis, OTUs (at 97 % identity threshold) with the same taxonomic classification were combined into a single bin, thereby generating a set of taxon bins where each bin had a unique taxonomy; we note here that the best taxonomic resolution for a taxon bin is at the genus level and that some of the bins may have less resolved taxonomy (that is, at one of phylum, class, order, or family levels). The sequence counts in these taxon bins were used to calculate their relative abundance in each sample, and these abundances were used as input features for the RF analysis. Gender, antibiotic use, and alpha diversity (Shannon index) were also included as input features.

## Results and discussion

### Cohort description

The patient cohort included 28 children and adolescent participants with ALL, age 3–23 years (median age 9.9 years), and consisted of 19 male and nine female members. Similarly, the control cohort included 23 sibling controls age 2–25 years (median age 9.3 years) with 16 females and seven males (Table 1). The patient cohort showed a high prevalence of ALL in males; it has been demonstrated previously that childhood cancer occurs more frequently in males than in females [30]. The mean age difference between patient and control groups was not statistically significant based on a *t*-test. A total of 180 fecal samples were collected and used over a period of one year for 16S rRNA sequencing and analysis (Additional file 1: Table S1). There were an average 16,270 annotated sequences per sample, (82,969 – maximum, and 10,498 – median). Four patients were positive for *C. difficile*. The low incidence of *C. difficile* in this immunocompromised patient population may reflect the efficiency of antibiotic prophylaxis and other mitigation strategies employed by the clinical care team.

### GI microbiota composition

**Fig. 1:**
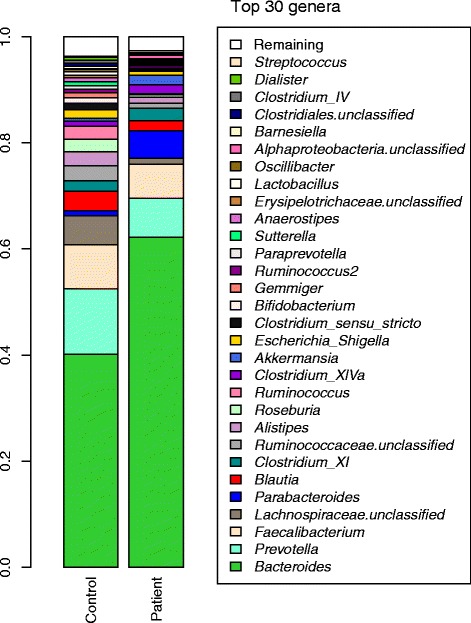
Mean microbial taxon abundances in the Patient and Control groups (Visit 1). 16S ribosomal RNA gene surveys in the stool samples are used to taxonomically identify the gut microbiota

The microbiota profiles (Visit 1) of both patient and control groups were dominated by members of the genera *Bacteroides*, *Prevotella*, and *Faecalibacterium*, with these having mean abundances of 62.2, 7.3, and 6.4 % respectively, in the patient group, and 40.2, 12.2, and 8.3 % respectively, in the control group. The rank orders of the remaining lower abundant taxa were different in the two groups (Fig. 1). However, at the genus level, both patient and control groups share many taxa in common (Fig. 1).

**Fig. 2:**
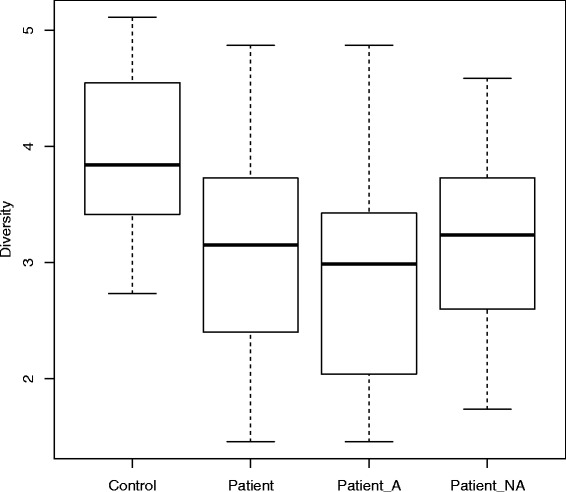
Box-plots of the alpha diversity (based on OTUs at 97 % indentity threshold) of the Control and Patient groups. The Patient group is further partitioned into the group taking antibiotics 1-month period Visit 1 (Patient_A) and the group not taking antibiotics (Patient_NA). The Y-axis denotes alpha diversity (Shannon Index values). The mean alpha-diversity values for the various groups are 3.92 (Control), 3.07 (Patient), 2.96 (Patient_A), and 3.25 (Patient_NA). The Patient group has a lower microbiota diversity (statistically significant) compared to the Control group (*p-value* = 0.0012). The diversities of the Patient_A and Patient_NA groups are also significantly lower (*p-value* < 0.05) than the Control group

The OTUs identified from the samples were used to compute the alpha diversity of the microbial communities (using the Shannon index). These calculations reveal the microbiota diversity (using OTUs at 97 % identity threshold) of the Patient group (Visit 1) to be lower than that of the Control group (Visit 1), with this difference being statistically significant (*p*-value 0.0012, Wilcoxon Rank Sum test) (Fig. 2). The Patient group was further partitioned into those who reported taking antibiotics in the one month period prior to Visit 1 (Patient_A group) and those who did not take antibiotics in this period (Patient_NA group). The microbiota diversity in each of these groups was compared with that of the Control group, and also found to be different using the Wilcoxon Rank Sum test *(p*-value for Control vs. Patient_A: 0.0027; *p*-value for Control vs. Patient_NA: 0.028). These observations also hold for diversity calculations based on OTUs generated using 95 and 90 % identity thresholds as well (Additional file 2: Figure S1).

**Fig. 3:**
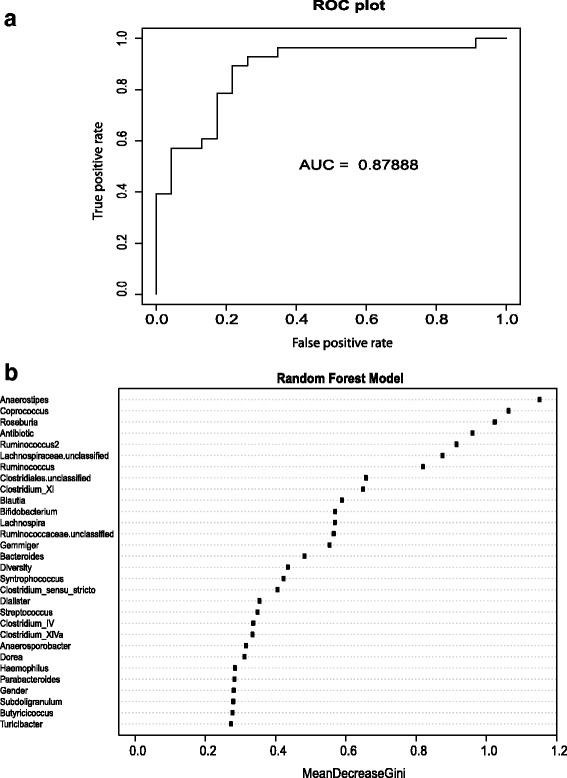
Random Forest analysis to distinguish between Patient and Control groups based on microbiota profiles (and sample metadata). The top figure Receiver Operating Characteristic (ROC) plot (**a**) shows the classification accuracy (as evaluated by AUC) while the bottom figure (**b**) lists the input features in decreasing order of importance (based on MeanDecreaseGini values)

It was possible to distinguish between the patient and control groups (Visit 1) based on their microbiota profiles. For this assessment, we used RF to identify features that could discriminate between the two groups. We observed a high classification accuracy, as measured by Area Under the Curve (AUC), of 87.9 % (Fig. 3). The input features (taxon abundances and sample metadata) were ranked by order of their importance in the classification (using their MeanDecreaseGini value). Based on this, the top taxa include *Anaerostipes*, *Coprococcus*, *Roseburia*, and *Ruminococcus2* (all of these being in higher relative abundance in the Control group), while metadata on antibiotic use and alpha diversity are also important.

The protective role of commensal intestinal bacteria in human disease is increasingly being appreciated. However, microbiome studies that investigate the role of bacteria in human disease have focused primarily on inflammatory bowel disease, which is caused by a chronic inflammatory process. Previous studies have shown that the intestinal microbiota in patients with inflammatory bowel disease (IBD) is characterized by a contraction of Firmicutes and Bacteroidetes and an expansion of Proteobacteria [31]. Lachnospiraceae (which comprises the *Clostridium* XIVa and IV groups within the order Clostridiales) and *Roseburia* (butyrate-producing bacterium) were greatly reduced, in acute leukemia patients compared to a healthy sibling (Fig. 1), but the *Bacteroides* are increased in patients compare to sibling controls. Bacteria producing butyrate play a major role in the composition of the mucus layer, as butyrate is an important energy source for intestinal epithelial cells and plays a role in the maintenance of colonic homeostasis [32]. Several intestinal bacteria produce short chain fatty acids (SCFAs), with butyrate being the most thoroughly investigated. Butyrate is produced by *F. prausnitzii* and *Clostridium* XIVa and has been shown to have profound anti-inflammatory effects [32–36]. The observed reduction in Lachnospiraceae and *Roseburia* in acute leukemia patients may increase the risk of developing chemotherapy induced mucositis and other GI complications in childhood leukemia patients.

### Microbiota diversity changes during chemotherapy and maintenance therapy

**Fig. 4:**
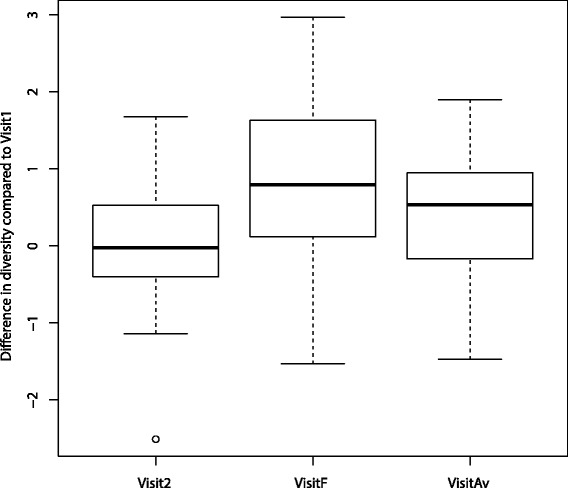
Microbiota diversity increases over the course of chemotherapy. Visit2 denotes the diversity difference between Visit 2 and Visit 1; VisitF denotes the diversity difference between the final visit and Visit 1; VisitAv denotes the difference in average diversity after Visit 1 and diversity at Visit 1. All three differences have mean values >0, and both VisitF and VisitAv are statistically significantly different (from 0)

We assessed the changes in microbiota diversity for the patients after chemotherapy (using OTUs calculated at 97 % identity threshold). As part of this, we evaluated differences in diversity for three cases: (a) diversity at Visit 2 (after chemotherapy) versus diversity at Visit 1 (before chemotherapy), (b) diversity at the final visit versus diversity at Visit 1, and (c) the average diversity for visits after Visit 1 versus diversity at Visit 1. For each of these comparisons, we used a paired (one sample) *t*-test to assess whether the difference in diversity was 0 or greater than 0. As shown in Fig. 4, while the diversity increase was not significant for case (a) with a *p*-value of 0.318, the increase for subsequent visits were significant, with *p*-values of 0.00026 and 0.00643 for cases (b) and (c) respectively.

It is likely that these observed changes in the GI microbiota diversity during therapy are the result of several factors including a direct influence of some of the therapeutic compounds or combination of chemotherapy and steroid prophylaxis on the gut flora, an indirect effect of chemotherapy on the immune system, which, in turn, affects the microbiome, or dietary choices during therapy. The lymphoid progenitor cells in ALL patients are affected during the disease, leading to the impaired immune system typically observed at the time of diagnosis [21, 37]. It is known that microbiome community structure is determined by both host and environmental factors [38]. If any one of these factors is greatly perturbed, a drastic composition shift in the composition of the microbiome can be expected.

## Conclusions

Several microbiome studies have demonstrated that administration of antibiotics can perturb this microflora temporarily and in particular cases permanently [12–15, 39]. Antibiotic-induced shifts can increase susceptibility to *C. difficile* infection [18]. Similarly, mucosal barrier injury, characterized by both inflammation and cell loss in the epithelial barrier lining of the gastrointestinal tract, is one of the most debilitating side effects of radiotherapy and chemotherapy treatment [39, 40]. However, the composition of GI microbiota in pediatric and adolescent leukemia patients and the microbiota changes after contemporary chemotherapy has not been investigated. Our study is the first, to the best of our knowledge, to address a population of pediatric and adolescent patients with acute leukemia and to compare these patients with their sibling controls. This characterization of the GI microbiota in pediatric and adolescent patients with acute leukemia has provided significant information on GI microbiota populations in immunocompromised individuals and opens up the potential for developing novel diagnostics based on stool tests as well as developing therapies to improve the dysbiotic condition of the microbiota at the time of diagnosis and in the earliest stages of chemotherapy. This creates the potential for the better management of GI and systemic complications associated with immunodeficiency and other disease conditions of this type. Furthermore, characterizing the GI microbiota dynamics and immune response following chemotherapy treatment will address what alterations happen to the GI microbiota during and following chemotherapy regimens and may be correlated with response to treatment. We anticipate expanding this study to get more detailed information on microbial profiles that are associated with or that lead to the development of various infections including *C. difficile* and persistent diarrhea. Further, we may find that the composition of the microbiota may ultimately be used as an indicator as to how well a patient may respond to different chemotherapy treatments.

## Abbreviations

ALL, acute lymphoblastic leukemia; GI, gastrointestinal; HMP, Human Microbiome Project; OTUs, Operational Taxonomic Units; PCR, polymerase chain reaction; RDP, Ribosomal Database Project; RF, Random Forests; rRNA, ribosomal ribonucleic acid.

## Additional files

Additional file 1: Table S1. List of fecal samples collected for each patient over the period of enrollment for 16S rRNA gene sequencing and analysis. (XLSX 17 kb) Additional file 2: Figure S1. Box-plots of the alpha diversity of OTUs at 95 % and 90 % identity threshold of the Control and Patient groups. The Patient group is further partitioned into the group taking antibiotics 1-month period Visit 1 (Patient_A) and the group not taking antibiotics (Patient_NA). (A) Alpha diversity for 95 % OTUs, the Y-axis denotes alpha diversity (Shannon Index values). The Wilcoxon Rank Sum test *p-value =* 0.00253 for Control vs Patient, *p-value* = 0.00328 for Control vs Patient_A and *p-value* = 0.05969 for Control vs Patient_NA. (B) Alpha diversity for 90 % OTUs, the Y-axis denotes alpha diversity (Shannon Index values). The Wilcoxon Rank Sum test *p-value* = 0.00156 for Control vs Patient, *p-value* = 0.00328 for Control vs Patient_A and *p-value* = 0.03119 for Control vs Patient_NA. In both cases (A, B) the Patient group has a lower microbiota diversity (statistically significant) compared to the Control group (*p-value* < 0.0026). The diversities of the Patient_A and Patient_NA groups are also significantly lower (*p-value* < 0.05) than the Control group. (PDF 203 kb)
