# Supplementary figures and images for: Gastrointestinal microbial populations can distinguish pediatric and adolescent Acute Lymphoblastic Leukemia (ALL) at the time of disease diagnosis
Source: BMC Genomics. 2016 Aug 15;17:635. doi: 10.1186/s12864-016-2965-y (PMC4986186; doi:10.1186/s12864-016-2965-y)

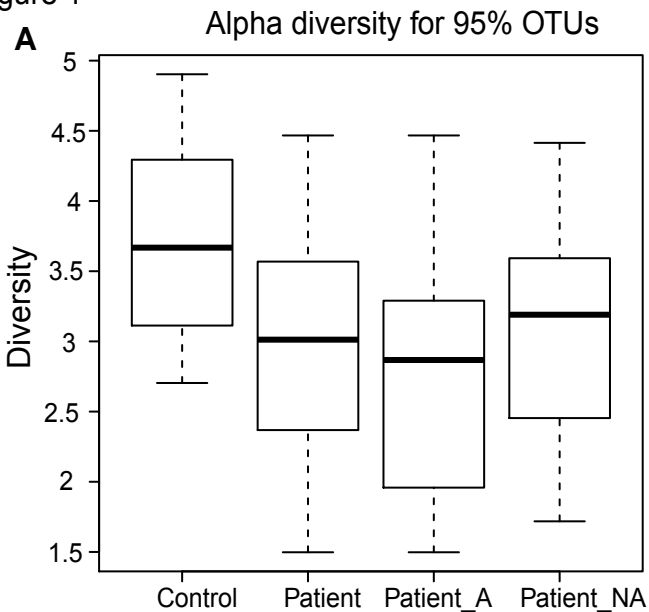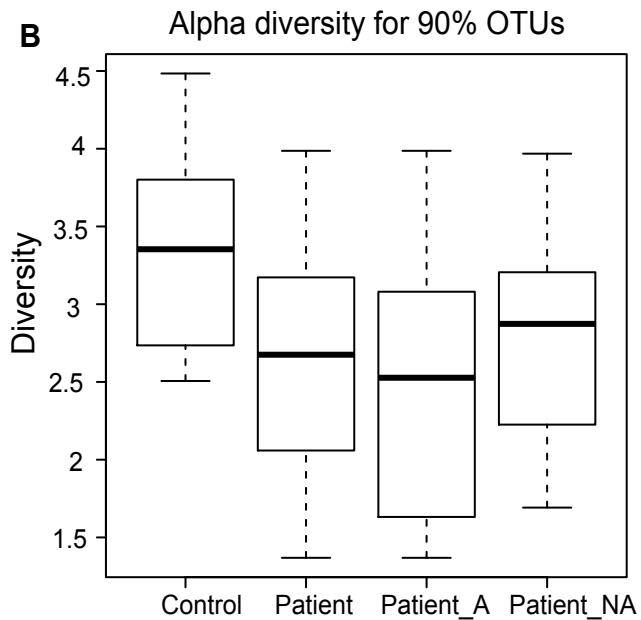

Supplement: Additional file 2: Figure S1. — Box-plots of the alpha diversity of OTUs at 95 % and 90 % identity threshold of the Control and Patient groups. The Patient group is further partitioned into the group taking antibiotics 1-month period Visit 1 (Patient_A) and the group not taking antibiotics (Patient_NA). (A) Alpha diversity for 95 % OTUs, the Y-axis denotes alpha diversity (Shannon Index values). The Wilcoxon Rank Sum test p-value = 0.00253 for Control vs Patient, p-value = 0.00328 for Control vs Patient_A and p-value = 0.05969 for Control vs Patient_NA. (B) Alpha diversity for 90 % OTUs, the Y-axis denotes alpha diversity (Shannon Index values). The Wilcoxon Rank Sum test p-value = 0.00156 for Control vs Patient, p-value = 0.00328 for Control vs Patient_A and p-value = 0.03119 for Control vs Patient_NA. In both cases (A, B) the Patient group has a lower microbiota diversity (statistically significant) compared to the Control group (p-value < 0.0026). The diversities of the Patient_A and Patient_NA groups are also significantly lower (p-value < 0.05) than the Control group. (PDF 203 kb) [file 12864_2016_2965_MOESM2_ESM.pdf]
